# Crystal structure of aqua­bis­[4-(methyl­sulfan­yl)benzoato-κ*O*](1,10-phenanthroline-κ^2^ *N*,*N*′)copper(II) monohydrate

**DOI:** 10.1107/S205698901501364X

**Published:** 2015-07-29

**Authors:** Jin-Li Zhu, Jian-hua Li, Miao Wang, Guo-Min Jiang, Guo-Qing Jiang

**Affiliations:** a College of Chemistry and Chemical Engineering, Nantong University, Nantong 226019, People’s Republic of China

**Keywords:** crystal structure, copper(II) complex, benzoate, 1,10-phenanthroline

## Abstract

In the title compound, [Cu(C_8_H_7_O_2_S)_2_(C_12_H_8_N_2_)(H_2_O)]·H_2_O, the central Cu^II^ atom is five-coordinated in a slightly distorted square-pyramidal environment by two N atoms from a 1,10-phenanthroline ligand, one O atom from the carboxylate group of one 4-(methyl­sulfan­yl)benzoate anion and one water O atom in the equatorial plane while the apical position is occupied by the O atom of a carboxylate group of the second anion. In the crystal, a three-dimensional supra­molecular network is formed through weak inter­molecular C—H⋯O and C—H⋯S inter­actions and π-stacking between the 1,10-phenanthroline ligand and the aromatic rings of symmetry-related 4-(methyl­sulfan­yl)benzoate ligands.

## Abstract 2

In a hydrated Cu^II^ complex with 1,10-phenanthroline and 4-(methyl­sulfan­yl)benzoate ligands, a three-dimensional supra­molecular network is formed through weak inter­molecular C—H⋯O and C—H⋯S inter­actions and π-stacking between the planes of phenanthroline and the aromatic rings of symmetry-related 4-(methyl­sulfan­yl)benzoate ligands.

## Chemical context   

There are numerous reasons for the rapidly increasing inter­est in the design and synthesis of metal-organic frameworks based on transition metal carboxyl­ate ligands. Not only do they often display fascinating structures in crystal engineering, but also have value due to their potential applications, including as homogeneous catalysts for various oxidation reactions (Bilgrien *et al.*, 1987 ▸; Zhang *et al.*, 2011 ▸), elucidation of electrical conductivity (Campbell *et al.*, 2015 ▸; Talin *et al.*, 2014 ▸), and as attractive mol­ecular magnetic materials (Kitagawa *et al.*, 2004 ▸; Janiak *et al.*, 2003 ▸). Transition metal complexes with thiol groups in their periphery are likely to play a vital role in the development of advanced functional materials because the functionalized thio­methyl groups around the periphery of the complex may provide binding sites for the surfaces of some specific materials, such as gold, silver, or palladium (Naitabdi *et al.*, 2005 ▸; Jiang *et al.*, 2014 ▸). As part of the above-mentioned systematic investigations, we report here the crystal structure of the title compound, Cu(OOCPhSCH_3_)_2_(N_2_C_12_H_12_)·H_2_O (I).
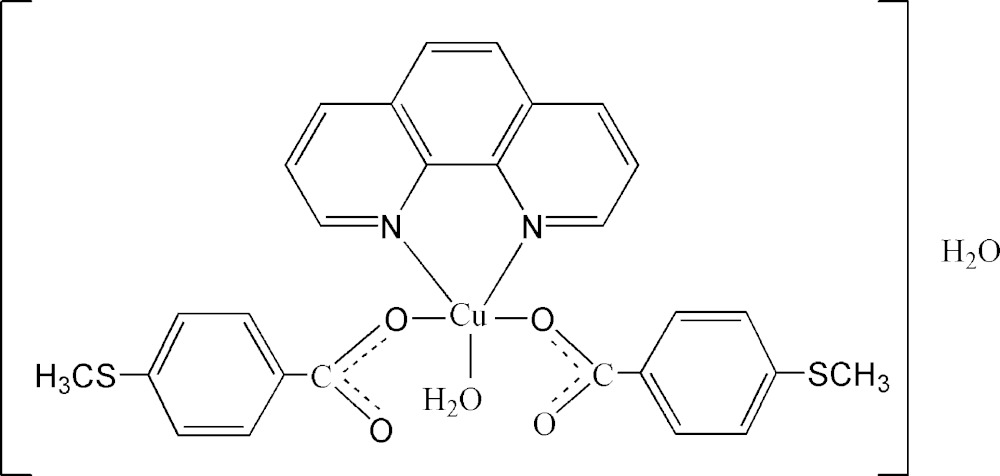


## Structure commentary   

**Figure 1:**
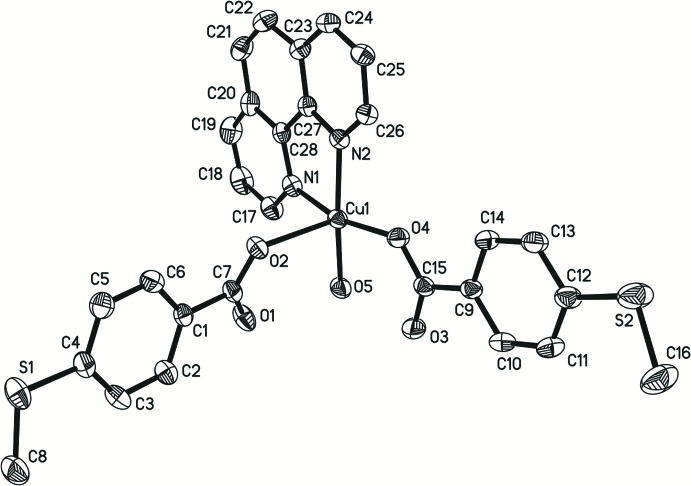
View of the coordination sphere around the Cu^II^ atom in the title compound, showing the atomic numbering scheme. Displacement ellipsoids are drawn at the 30% probability level.

In (I), the central Cu^II^ atom has a slightly distorted square-pyramidal coordination geometry (Fig. 1 ▸). The equatorial plane is formed by two nitro­gen atoms from the 1,10-phenanthroline ligand, one oxygen atom from the carboxyl­ate group of a 4-(methyl­sulfan­yl)benzoate anion and one water oxygen atom, whereas the apical position is occupied by a carboxylate O atom from the second anion. The average Cu—N bond length is 2.014 (6) Å, the Cu—O(carboxyl­ate) bond length is 1.945 (2) Å, while the Cu—O(water) is 1.953 (2) Å. The apical Cu—O distance is 2.301 (2) Å. Two intra­molecular hydrogen bonds involving the coordinating water mol­ecule, O5—H5*A*⋯O3 and O5—H5*B*⋯O1, are observed (Table 1 ▸).

## Supra­molecular features   

**Figure 2:**
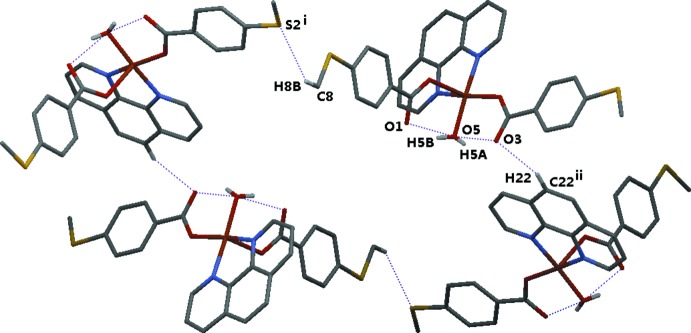
C—H⋯O and C—H⋯S hydrogen-bonding inter­actions in (I) [symmetry codes: (i) 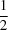 + *x*, 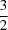 − *y*, 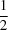 + *z*; (ii) 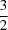 − *x*, 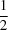 + *y*, 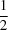 − *z*]. H atoms and water mol­ecules have been omitted for clarity.

**Figure 3:**
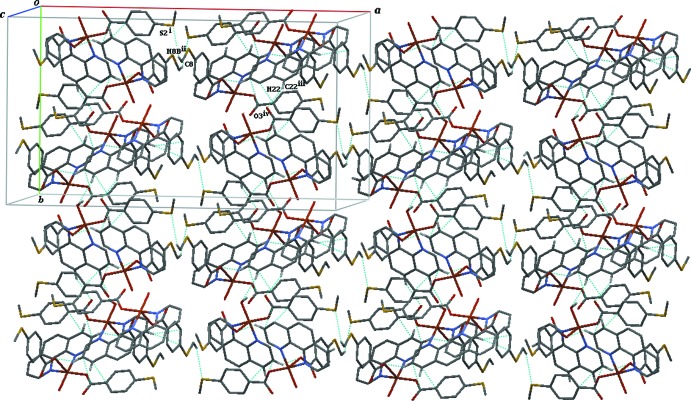
Projection along the *c* axis of the three-dimensional framework in (I), showing the cavities. H atoms and water mol­ecules have been omitted for clarity. [Symmetry codes: (i) −*x* + 1, −*y* + 1, −*z* + 1; (ii) −*x* + 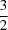, *y* − 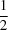, −*z* + 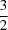; (iii) *x*, −*y* + 1, *z* + 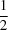; (iv) −*x* + 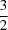, −*y* + 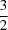, −*z* + 1.]

In the crystal, the complex mol­ecules are linked into a supramolecular framework (Fig. 2 ▸) by significant offset C—H⋯O and C—H⋯S hydrogen bonds (see Table 1 ▸). The complex mol­ecule is linked to the solvent water mol­ecule by an O—H⋯O hydrogen bond. The overall three-dimensional supra­molecular structure is also stabilized by π-stacking between the 1,10-phenanthroline ligands and the aromatic rings of 4-(methyl­sulfan­yl)benzoic acid of symmetry-related mol­ecules (Fig. 3 ▸).

## Synthesis and Crystallization   

Copper(II) acetate monohydrate (0.1997 g, 1 mmol) in H_2_O (10 mL) was added to a stirred solution of the sodium salt of 4-(methyl­sulfan­yl)benzoic acid (0.19 g, 1 mmol) in H_2_O (10 mL) and phenanthroline (0.18 g, 1 mmol) in anhydrous alcohol (10 mL). The mixture was then stirred for two h, and then filtrated. Single crystals of the title complex were obtained by slow evaporation of this filtrate.

## Refinement   

Crystal data, data collection and structure refinement details are summarized in Table 2 ▸. Carbon-bound H atoms were positioned geometrically, with C—H = 0.97 Å for methyl­ene and 0.93 Å for aromatic, and refined using a riding model, with *U*_iso_ (H) = 1.2 *U*_eq_(C). The water H atoms were located from difference maps and refined with *d*(O—H) = 0.79 Å and *U*_iso_(H) = 1.5*U*_eq_(O) for the coordinating water molecule, and with *d*(O—H) = 0.85 Å and *U*_iso_(H) = 1.5*U*_eq_(O) for the solvent water molecule. The hydroxyl H atom was positioned geometrically and freely refined.

## Supplementary Material

Crystal structure: contains datablock(s) global, I. DOI: 10.1107/S205698901501364X/gw2152sup1.cif

Structure factors: contains datablock(s) I. DOI: 10.1107/S205698901501364X/gw2152Isup2.hkl

CCDC reference: 1413518

Additional supporting information:  crystallographic information; 3D view; checkCIF report

## Figures and Tables

**Table 1:** Hydrogen-bond geometry (, )

*D*H*A*	*D*H	H*A*	*D* *A*	*D*H*A*
O5H5*A*O3	0.79	1.90	2.614(3)	149
O5H5*B*O1	0.79	1.82	2.554(3)	154
C8H8*B*S2^i^	0.96	2.97	3.672(5)	131
O6H6*B*O1^ii^	0.85	2.06	2.845(4)	154
C18H18O6^iii^	0.93	2.45	3.361(6)	168
C22H22O3^iv^	0.93	2.51	3.364(4)	153

Symmetry codes: (i) 
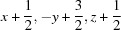
; (ii) 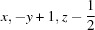; (iii) 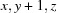; (iv) 
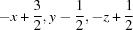
.

**Table 2:** Experimental details

Crystal data	
Chemical formula	[Cu(C_8_H_7_O_2_S)_2_(C_12_H_8_N_2_)(H_2_O)]H_2_O
*M*_r_	614.18
Crystal system, space group	Monoclinic, *C*2/*c*
Temperature (K)	293
*a*, *b*, *c* ()	30.2105(12), 17.2468(6), 10.7009(4)
()	101.426(2)
*V* (^3^)	5465.0(4)
*Z*	8
Radiation type	Mo *K*
(mm^1^)	1.00
Crystal size (mm)	0.23 0.18 0.13

Data collection	
Diffractometer	Bruker SMART APEX CCD area detector
Absorption correction	Multi-scan (*SADABS*; Bruker, 2000 ▸)
*T*_min_, *T*_max_	0.879, 0.956
No. of measured, independent and observed [*I* > 2(*I*)] reflections	24423, 6357, 4364
*R*_int_	0.033
(sin /)_max_ (^1^)	0.652

Refinement	
*R*[*F* ^2^ > 2(*F* ^2^)], *wR*(*F* ^2^), *S*	0.050, 0.178, 1.04
No. of reflections	6357
No. of parameters	354
H-atom treatment	H-atom parameters constrained
_max_, _min_ (e ^3^)	0.42, 0.67

Computer programs: *APEX2* and *SAINT* (Bruker, 2000 ▸), *SHELXS97*, *SHELXL97* and *SHELXTL* (Sheldrick, 2008 ▸).

## supplementary crystallographic information

### Crystal data

**Table:**

[Cu(C_8_H_7_O_2_S)_2_(C_12_H_8_N_2_)(H_2_O)]·H_2_O	*Z* = 8
*M**_r_* = 614.18	*F*(000) = 2536
Monoclinic, *C*2/*c*	*D*_x_ = 1.493 Mg m^−^^3^
Hall symbol: -C 2yc	Mo *K*α radiation, λ = 0.71073 Å
*a* = 30.2105 (12) Å	θ = 3.2–24.5°
*b* = 17.2468 (6) Å	µ = 1.00 mm^−^^1^
*c* = 10.7009 (4) Å	*T* = 293 K
β = 101.426 (2)°	Block, blue
*V* = 5465.0 (4) Å^3^	0.23 × 0.18 × 0.13 mm

### Data collection

**Table:**

Bruker SMART APEX CCD area-detector diffractometer	6357 independent reflections
Radiation source: fine-focus sealed tube	4364 reflections with *I* > 2σ(*I*)
Graphite monochromator	*R*_int_ = 0.033
phi and ω scans	θ_max_ = 27.6°, θ_min_ = 2.3°
Absorption correction: multi-scan (*SADABS*; Bruker, 2000)	*h* = −39→37
*T*_min_ = 0.879, *T*_max_ = 0.956	*k* = −18→22
24423 measured reflections	*l* = −13→13

### Refinement

**Table:**

Refinement on *F*^2^	Primary atom site location: structure-invariant direct methods
Least-squares matrix: full	Secondary atom site location: difference Fourier map
*R*[*F*^2^ > 2σ(*F*^2^)] = 0.050	Hydrogen site location: inferred from neighbouring sites
*wR*(*F*^2^) = 0.178	H-atom parameters constrained
*S* = 1.04	*w* = 1/[σ^2^(*F*_o_^2^) + (0.1167*P*)^2^] where *P* = (*F*_o_^2^ + 2*F*_c_^2^)/3
6357 reflections	(Δ/σ)_max_ = 0.001
354 parameters	Δρ_max_ = 0.42 e Å^−^^3^
0 restraints	Δρ_min_ = −0.67 e Å^−^^3^

### Special details

**Table:**

Geometry. All e.s.d.'s (except the e.s.d. in the dihedral angle between two l.s. planes) are estimated using the full covariance matrix. The cell e.s.d.'s are taken into account individually in the estimation of e.s.d.'s in distances, angles and torsion angles; correlations between e.s.d.'s in cell parameters are only used when they are defined by crystal symmetry. An approximate (isotropic) treatment of cell e.s.d.'s is used for estimating e.s.d.'s involving l.s. planes.
Refinement. Refinement of *F*^2^ against ALL reflections. The weighted *R*-factor *wR* and goodness of fit *S* are based on *F*^2^, conventional *R*-factors *R* are based on *F*, with *F* set to zero for negative *F*^2^. The threshold expression of *F*^2^ > σ(*F*^2^) is used only for calculating *R*-factors(gt) *etc*. and is not relevant to the choice of reflections for refinement. *R*-factors based on *F*^2^ are statistically about twice as large as those based on *F*, and *R*- factors based on ALL data will be even larger.

### Fractional atomic coordinates and isotropic or equivalent isotropic displacement parameters (Å^2^)

**Table:**

	*x*	*y*	*z*	*U*_iso_*/*U*_eq_	
C1	0.91367 (10)	0.80765 (19)	0.6949 (3)	0.0395 (7)	
C2	0.94361 (12)	0.8504 (2)	0.7863 (3)	0.0473 (8)	
H2	0.9447	0.9040	0.7783	0.057*	
C3	0.97159 (12)	0.8147 (2)	0.8880 (3)	0.0507 (9)	
H3	0.9914	0.8443	0.9469	0.061*	
C4	0.97032 (12)	0.7360 (2)	0.9025 (3)	0.0487 (8)	
C5	0.94020 (15)	0.6925 (2)	0.8154 (4)	0.0624 (10)	
H5	0.9388	0.6391	0.8258	0.075*	
C6	0.91192 (14)	0.7280 (2)	0.7125 (3)	0.0568 (9)	
H6	0.8917	0.6981	0.6552	0.068*	
C7	0.88454 (10)	0.8481 (2)	0.5835 (3)	0.0420 (7)	
C8	1.03311 (16)	0.7601 (3)	1.1220 (4)	0.0782 (13)	
H8A	1.0536	0.7839	1.0756	0.117*	
H8B	1.0498	0.7390	1.2004	0.117*	
H8C	1.0121	0.7982	1.1404	0.117*	
C9	0.70228 (11)	0.94306 (18)	0.6029 (3)	0.0395 (7)	
C10	0.69449 (13)	0.99837 (19)	0.6908 (3)	0.0464 (8)	
H10	0.7150	1.0386	0.7133	0.056*	
C11	0.65679 (13)	0.9942 (2)	0.7449 (3)	0.0530 (9)	
H11	0.6518	1.0322	0.8023	0.064*	
C12	0.62624 (12)	0.9342 (2)	0.7147 (3)	0.0515 (9)	
C13	0.63413 (13)	0.8776 (2)	0.6289 (3)	0.0516 (9)	
H13	0.6141	0.8365	0.6085	0.062*	
C14	0.67156 (13)	0.88267 (18)	0.5744 (3)	0.0458 (8)	
H14	0.6764	0.8447	0.5169	0.055*	
C15	0.74312 (11)	0.94912 (18)	0.5455 (3)	0.0398 (7)	
C16	0.58169 (19)	0.9886 (3)	0.9050 (4)	0.0901 (16)	
H16A	0.6090	0.9776	0.9655	0.135*	
H16B	0.5562	0.9829	0.9455	0.135*	
H16C	0.5827	1.0407	0.8743	0.135*	
C17	0.86956 (12)	0.9017 (2)	0.2168 (4)	0.0549 (9)	
H17	0.8782	0.9471	0.2621	0.066*	
C18	0.89325 (14)	0.8779 (3)	0.1231 (4)	0.0674 (12)	
H18	0.9170	0.9079	0.1068	0.081*	
C19	0.88197 (13)	0.8122 (3)	0.0567 (4)	0.0629 (11)	
H19	0.8981	0.7967	−0.0046	0.076*	
C20	0.84605 (12)	0.7673 (2)	0.0800 (3)	0.0475 (8)	
C21	0.83066 (14)	0.6981 (2)	0.0145 (3)	0.0549 (9)	
H21	0.8461	0.6786	−0.0456	0.066*	
C22	0.79377 (13)	0.6595 (2)	0.0372 (3)	0.0525 (9)	
H22	0.7843	0.6146	−0.0083	0.063*	
C23	0.76929 (11)	0.68729 (18)	0.1307 (3)	0.0416 (7)	
C24	0.73153 (12)	0.65166 (19)	0.1591 (3)	0.0447 (8)	
H24	0.7201	0.6068	0.1162	0.054*	
C25	0.71115 (12)	0.68223 (19)	0.2497 (3)	0.0445 (8)	
H25	0.6854	0.6591	0.2679	0.053*	
C26	0.72889 (11)	0.74829 (18)	0.3155 (3)	0.0400 (7)	
H26	0.7148	0.7679	0.3786	0.048*	
C27	0.78475 (10)	0.75391 (17)	0.1992 (3)	0.0362 (7)	
C28	0.82339 (10)	0.79504 (18)	0.1740 (3)	0.0378 (7)	
H5B	0.8518	0.9712	0.4639	0.045*	
Cu1	0.797423 (13)	0.87819 (2)	0.37570 (3)	0.03815 (16)	
N1	0.83506 (9)	0.86080 (16)	0.2421 (3)	0.0430 (6)	
N2	0.76492 (8)	0.78433 (15)	0.2920 (2)	0.0368 (6)	
O1	0.89755 (8)	0.91478 (15)	0.5570 (2)	0.0581 (7)	
O2	0.85009 (8)	0.81458 (13)	0.5254 (2)	0.0458 (5)	
O3	0.76917 (8)	1.00411 (14)	0.5740 (3)	0.0558 (6)	
O4	0.74829 (8)	0.89487 (13)	0.4657 (2)	0.0439 (5)	
O5	0.82673 (8)	0.97740 (12)	0.4260 (2)	0.0489 (6)	
H5A	0.8159	0.9985	0.4786	0.073*	
O6	0.97937 (10)	−0.00119 (17)	0.1092 (3)	0.0822 (9)	
H6B	0.9609	0.0363	0.0910	0.099*	
H6C	1.0066	0.0119	0.1101	0.099*	
S1	1.00341 (4)	0.68523 (7)	1.02966 (10)	0.0694 (3)	
S2	0.57647 (4)	0.92277 (9)	0.77475 (13)	0.0935 (5)	

### Atomic displacement parameters (Å^2^)

**Table:**

	*U*^11^	*U*^22^	*U*^33^	*U*^12^	*U*^13^	*U*^23^
C1	0.0325 (16)	0.0469 (19)	0.0395 (16)	0.0020 (13)	0.0083 (13)	0.0057 (13)
C2	0.0399 (19)	0.0486 (19)	0.0525 (19)	−0.0084 (15)	0.0070 (15)	0.0088 (15)
C3	0.0421 (19)	0.067 (2)	0.0403 (17)	−0.0142 (17)	0.0018 (15)	0.0081 (16)
C4	0.0395 (19)	0.066 (2)	0.0411 (17)	0.0069 (16)	0.0082 (15)	0.0065 (16)
C5	0.073 (3)	0.044 (2)	0.064 (2)	0.0081 (19)	−0.002 (2)	0.0067 (17)
C6	0.059 (2)	0.052 (2)	0.052 (2)	0.0027 (18)	−0.0051 (18)	−0.0054 (16)
C7	0.0313 (16)	0.054 (2)	0.0401 (16)	−0.0002 (15)	0.0059 (13)	0.0022 (14)
C8	0.077 (3)	0.102 (4)	0.048 (2)	0.018 (3)	−0.006 (2)	0.002 (2)
C9	0.0458 (18)	0.0377 (17)	0.0316 (15)	0.0016 (14)	−0.0005 (13)	−0.0005 (12)
C10	0.059 (2)	0.0433 (18)	0.0351 (16)	−0.0090 (15)	0.0050 (15)	−0.0059 (13)
C11	0.066 (2)	0.053 (2)	0.0409 (17)	−0.0049 (18)	0.0134 (17)	−0.0128 (15)
C12	0.053 (2)	0.064 (2)	0.0388 (17)	−0.0093 (17)	0.0111 (16)	−0.0106 (15)
C13	0.053 (2)	0.053 (2)	0.0487 (19)	−0.0111 (16)	0.0089 (17)	−0.0131 (15)
C14	0.051 (2)	0.0438 (19)	0.0411 (17)	−0.0023 (15)	0.0056 (15)	−0.0104 (14)
C15	0.0417 (18)	0.0358 (16)	0.0376 (16)	0.0002 (14)	−0.0026 (14)	0.0021 (13)
C16	0.107 (4)	0.097 (4)	0.080 (3)	−0.016 (3)	0.051 (3)	−0.034 (3)
C17	0.046 (2)	0.054 (2)	0.063 (2)	−0.0111 (17)	0.0098 (18)	0.0098 (18)
C18	0.047 (2)	0.082 (3)	0.078 (3)	−0.010 (2)	0.024 (2)	0.023 (2)
C19	0.055 (2)	0.072 (3)	0.070 (2)	0.003 (2)	0.034 (2)	0.013 (2)
C20	0.0464 (19)	0.052 (2)	0.0472 (18)	0.0092 (16)	0.0162 (16)	0.0163 (15)
C21	0.067 (2)	0.056 (2)	0.0467 (18)	0.0110 (18)	0.0229 (18)	0.0013 (16)
C22	0.073 (3)	0.0426 (19)	0.0441 (18)	0.0016 (18)	0.0170 (18)	−0.0016 (15)
C23	0.050 (2)	0.0378 (17)	0.0369 (15)	0.0023 (14)	0.0097 (14)	0.0034 (13)
C24	0.055 (2)	0.0387 (17)	0.0386 (16)	−0.0060 (15)	0.0049 (15)	−0.0007 (13)
C25	0.0444 (19)	0.0447 (18)	0.0439 (17)	−0.0101 (14)	0.0075 (15)	0.0037 (14)
C26	0.0414 (18)	0.0430 (17)	0.0366 (15)	−0.0035 (14)	0.0100 (14)	0.0003 (13)
C27	0.0387 (17)	0.0391 (16)	0.0302 (14)	0.0035 (13)	0.0052 (12)	0.0088 (12)
C28	0.0386 (17)	0.0384 (16)	0.0356 (15)	0.0036 (13)	0.0053 (13)	0.0109 (12)
Cu1	0.0367 (2)	0.0361 (2)	0.0400 (2)	−0.00390 (15)	0.00356 (17)	0.00118 (15)
N1	0.0383 (15)	0.0448 (15)	0.0454 (15)	−0.0032 (12)	0.0068 (12)	0.0104 (12)
N2	0.0373 (14)	0.0416 (14)	0.0314 (12)	−0.0016 (11)	0.0065 (11)	0.0024 (10)
O1	0.0417 (14)	0.0576 (16)	0.0684 (16)	−0.0104 (12)	−0.0048 (12)	0.0218 (12)
O2	0.0392 (13)	0.0479 (13)	0.0464 (12)	−0.0033 (10)	−0.0008 (10)	0.0060 (10)
O3	0.0497 (15)	0.0448 (14)	0.0723 (16)	−0.0081 (11)	0.0101 (13)	−0.0149 (12)
O4	0.0457 (13)	0.0413 (12)	0.0445 (12)	−0.0041 (10)	0.0083 (10)	−0.0057 (9)
O5	0.0430 (13)	0.0382 (12)	0.0610 (15)	−0.0046 (10)	−0.0003 (11)	0.0019 (10)
O6	0.0511 (17)	0.0655 (18)	0.134 (3)	−0.0018 (14)	0.0276 (18)	−0.0165 (17)
S1	0.0648 (7)	0.0801 (7)	0.0561 (6)	0.0082 (5)	−0.0055 (5)	0.0215 (5)
S2	0.0787 (8)	0.1247 (12)	0.0894 (9)	−0.0404 (8)	0.0461 (7)	−0.0575 (8)

### Geometric parameters (Å, º)

**Table:**

C1—C6	1.389 (5)	C16—H16C	0.9600
C1—C2	1.403 (5)	C17—N1	1.330 (4)
C1—C7	1.505 (4)	C17—C18	1.403 (6)
C2—C3	1.384 (5)	C17—H17	0.9300
C2—H2	0.9300	C18—C19	1.345 (6)
C3—C4	1.366 (5)	C18—H18	0.9300
C3—H3	0.9300	C19—C20	1.395 (5)
C4—C5	1.387 (5)	C19—H19	0.9300
C4—S1	1.755 (4)	C20—C28	1.408 (4)
C5—C6	1.395 (5)	C20—C21	1.415 (5)
C5—H5	0.9300	C21—C22	1.361 (5)
C6—H6	0.9300	C21—H21	0.9300
C7—O2	1.243 (4)	C22—C23	1.439 (4)
C7—O1	1.266 (4)	C22—H22	0.9300
C8—S1	1.759 (5)	C23—C24	1.382 (4)
C8—H8A	0.9600	C23—C27	1.392 (4)
C8—H8B	0.9600	C24—C25	1.354 (4)
C8—H8C	0.9600	C24—H24	0.9300
C9—C14	1.388 (4)	C25—C26	1.390 (4)
C9—C10	1.392 (4)	C25—H25	0.9300
C9—C15	1.487 (5)	C26—N2	1.320 (4)
C10—C11	1.378 (5)	C26—H26	0.9300
C10—H10	0.9300	C27—N2	1.363 (4)
C11—C12	1.382 (5)	C27—C28	1.436 (4)
C11—H11	0.9300	C28—N1	1.356 (4)
C12—C13	1.392 (5)	Cu1—O4	1.945 (2)
C12—S2	1.759 (4)	Cu1—O5	1.953 (2)
C13—C14	1.374 (5)	Cu1—N2	2.010 (3)
C13—H13	0.9300	Cu1—N1	2.018 (3)
C14—H14	0.9300	Cu1—O2	2.301 (2)
C15—O3	1.232 (4)	O5—H5B	0.7928
C15—O4	1.296 (4)	O5—H5A	0.7938
C16—S2	1.780 (4)	O6—H6B	0.8499
C16—H16A	0.9600	O6—H6C	0.8500
C16—H16B	0.9600		
			
C6—C1—C2	117.5 (3)	C19—C18—C17	120.8 (3)
C6—C1—C7	122.2 (3)	C19—C18—H18	119.6
C2—C1—C7	120.3 (3)	C17—C18—H18	119.6
C3—C2—C1	121.6 (3)	C18—C19—C20	119.8 (4)
C3—C2—H2	119.2	C18—C19—H19	120.1
C1—C2—H2	119.2	C20—C19—H19	120.1
C4—C3—C2	120.3 (3)	C19—C20—C28	116.4 (3)
C4—C3—H3	119.8	C19—C20—C21	124.8 (3)
C2—C3—H3	119.8	C28—C20—C21	118.8 (3)
C3—C4—C5	119.4 (3)	C22—C21—C20	121.6 (3)
C3—C4—S1	124.0 (3)	C22—C21—H21	119.2
C5—C4—S1	116.6 (3)	C20—C21—H21	119.2
C4—C5—C6	120.7 (4)	C21—C22—C23	120.9 (3)
C4—C5—H5	119.7	C21—C22—H22	119.6
C6—C5—H5	119.7	C23—C22—H22	119.6
C1—C6—C5	120.5 (4)	C24—C23—C27	117.2 (3)
C1—C6—H6	119.8	C24—C23—C22	124.4 (3)
C5—C6—H6	119.8	C27—C23—C22	118.4 (3)
O2—C7—O1	125.2 (3)	C25—C24—C23	119.8 (3)
O2—C7—C1	118.7 (3)	C25—C24—H24	120.1
O1—C7—C1	116.1 (3)	C23—C24—H24	120.1
S1—C8—H8A	109.5	C24—C25—C26	119.8 (3)
S1—C8—H8B	109.5	C24—C25—H25	120.1
H8A—C8—H8B	109.5	C26—C25—H25	120.1
S1—C8—H8C	109.5	N2—C26—C25	122.6 (3)
H8A—C8—H8C	109.5	N2—C26—H26	118.7
H8B—C8—H8C	109.5	C25—C26—H26	118.7
C14—C9—C10	117.9 (3)	N2—C27—C23	123.4 (3)
C14—C9—C15	122.4 (3)	N2—C27—C28	115.9 (3)
C10—C9—C15	119.7 (3)	C23—C27—C28	120.6 (3)
C11—C10—C9	120.8 (3)	N1—C28—C20	123.8 (3)
C11—C10—H10	119.6	N1—C28—C27	116.6 (3)
C9—C10—H10	119.6	C20—C28—C27	119.6 (3)
C10—C11—C12	120.7 (3)	O4—Cu1—O5	94.74 (10)
C10—C11—H11	119.7	O4—Cu1—N2	89.22 (9)
C12—C11—H11	119.7	O5—Cu1—N2	169.62 (9)
C11—C12—C13	119.0 (3)	O4—Cu1—N1	164.97 (11)
C11—C12—S2	125.3 (3)	O5—Cu1—N1	92.14 (11)
C13—C12—S2	115.7 (3)	N2—Cu1—N1	81.88 (10)
C14—C13—C12	119.9 (3)	O4—Cu1—O2	102.47 (9)
C14—C13—H13	120.1	O5—Cu1—O2	90.72 (9)
C12—C13—H13	120.1	N2—Cu1—O2	97.80 (9)
C13—C14—C9	121.7 (3)	N1—Cu1—O2	90.77 (10)
C13—C14—H14	119.2	C17—N1—C28	117.7 (3)
C9—C14—H14	119.2	C17—N1—Cu1	129.8 (3)
O3—C15—O4	124.4 (3)	C28—N1—Cu1	112.4 (2)
O3—C15—C9	119.6 (3)	C26—N2—C27	117.1 (3)
O4—C15—C9	116.0 (3)	C26—N2—Cu1	130.0 (2)
S2—C16—H16A	109.5	C27—N2—Cu1	112.9 (2)
S2—C16—H16B	109.5	C7—O2—Cu1	121.4 (2)
H16A—C16—H16B	109.5	C15—O4—Cu1	129.5 (2)
S2—C16—H16C	109.5	Cu1—O5—H5B	111.0
H16A—C16—H16C	109.5	Cu1—O5—H5A	111.7
H16B—C16—H16C	109.5	H5B—O5—H5A	100.8
N1—C17—C18	121.4 (4)	H6B—O6—H6C	112.9
N1—C17—H17	119.3	C4—S1—C8	102.6 (2)
C18—C17—H17	119.3	C12—S2—C16	105.4 (2)
			
C6—C1—C2—C3	2.2 (5)	N2—C27—C28—N1	−2.1 (4)
C7—C1—C2—C3	−178.4 (3)	C23—C27—C28—N1	177.7 (3)
C1—C2—C3—C4	−0.6 (5)	N2—C27—C28—C20	179.7 (3)
C2—C3—C4—C5	−1.1 (5)	C23—C27—C28—C20	−0.5 (4)
C2—C3—C4—S1	−178.6 (3)	C18—C17—N1—C28	−0.1 (5)
C3—C4—C5—C6	1.2 (6)	C18—C17—N1—Cu1	176.6 (3)
S1—C4—C5—C6	178.9 (3)	C20—C28—N1—C17	1.0 (5)
C2—C1—C6—C5	−2.1 (5)	C27—C28—N1—C17	−177.2 (3)
C7—C1—C6—C5	178.6 (3)	C20—C28—N1—Cu1	−176.3 (2)
C4—C5—C6—C1	0.4 (6)	C27—C28—N1—Cu1	5.5 (3)
C6—C1—C7—O2	19.7 (5)	O4—Cu1—N1—C17	123.6 (4)
C2—C1—C7—O2	−159.7 (3)	O5—Cu1—N1—C17	6.4 (3)
C6—C1—C7—O1	−160.6 (3)	N2—Cu1—N1—C17	177.9 (3)
C2—C1—C7—O1	20.0 (4)	O2—Cu1—N1—C17	−84.4 (3)
C14—C9—C10—C11	−1.7 (5)	O4—Cu1—N1—C28	−59.5 (5)
C15—C9—C10—C11	179.7 (3)	O5—Cu1—N1—C28	−176.7 (2)
C9—C10—C11—C12	1.2 (5)	N2—Cu1—N1—C28	−5.3 (2)
C10—C11—C12—C13	0.2 (6)	O2—Cu1—N1—C28	92.5 (2)
C10—C11—C12—S2	−179.0 (3)	C25—C26—N2—C27	−0.2 (5)
C11—C12—C13—C14	−0.9 (6)	C25—C26—N2—Cu1	−178.5 (2)
S2—C12—C13—C14	178.4 (3)	C23—C27—N2—C26	−0.8 (4)
C12—C13—C14—C9	0.3 (6)	C28—C27—N2—C26	178.9 (3)
C10—C9—C14—C13	1.0 (5)	C23—C27—N2—Cu1	177.7 (2)
C15—C9—C14—C13	179.6 (3)	C28—C27—N2—Cu1	−2.5 (3)
C14—C9—C15—O3	179.8 (3)	O4—Cu1—N2—C26	−9.6 (3)
C10—C9—C15—O3	−1.7 (5)	O5—Cu1—N2—C26	−122.2 (5)
C14—C9—C15—O4	0.6 (4)	N1—Cu1—N2—C26	−177.5 (3)
C10—C9—C15—O4	179.1 (3)	O2—Cu1—N2—C26	92.9 (3)
N1—C17—C18—C19	−0.7 (6)	O4—Cu1—N2—C27	172.0 (2)
C17—C18—C19—C20	0.6 (6)	O5—Cu1—N2—C27	59.5 (6)
C18—C19—C20—C28	0.2 (6)	N1—Cu1—N2—C27	4.2 (2)
C18—C19—C20—C21	178.7 (4)	O2—Cu1—N2—C27	−85.5 (2)
C19—C20—C21—C22	−176.3 (4)	O1—C7—O2—Cu1	−9.7 (4)
C28—C20—C21—C22	2.2 (5)	C1—C7—O2—Cu1	170.0 (2)
C20—C21—C22—C23	−0.9 (6)	O4—Cu1—O2—C7	−105.1 (2)
C21—C22—C23—C24	179.8 (3)	O5—Cu1—O2—C7	−10.1 (2)
C21—C22—C23—C27	−1.2 (5)	N2—Cu1—O2—C7	164.0 (2)
C27—C23—C24—C25	0.4 (5)	N1—Cu1—O2—C7	82.1 (2)
C22—C23—C24—C25	179.5 (3)	O3—C15—O4—Cu1	3.7 (5)
C23—C24—C25—C26	−1.4 (5)	C9—C15—O4—Cu1	−177.1 (2)
C24—C25—C26—N2	1.3 (5)	O5—Cu1—O4—C15	−9.0 (3)
C24—C23—C27—N2	0.7 (5)	N2—Cu1—O4—C15	−179.4 (3)
C22—C23—C27—N2	−178.4 (3)	N1—Cu1—O4—C15	−126.0 (4)
C24—C23—C27—C28	−179.0 (3)	O2—Cu1—O4—C15	82.8 (3)
C22—C23—C27—C28	1.8 (5)	C3—C4—S1—C8	1.7 (4)
C19—C20—C28—N1	−1.0 (5)	C5—C4—S1—C8	−175.8 (3)
C21—C20—C28—N1	−179.6 (3)	C11—C12—S2—C16	−13.4 (4)
C19—C20—C28—C27	177.1 (3)	C13—C12—S2—C16	167.4 (3)
C21—C20—C28—C27	−1.5 (5)		

### Hydrogen-bond geometry (Å, º)

**Table:**

*D*—H···*A*	*D*—H	H···*A*	*D*···*A*	*D*—H···*A*
O5—H5*A*···O3	0.79	1.90	2.614 (3)	149
O5—H5*B*···O1	0.79	1.82	2.554 (3)	154
C8—H8*B*···S2^i^	0.96	2.97	3.672 (5)	131
O6—H6*B*···O1^ii^	0.85	2.06	2.845 (4)	154
C18—H18···O6^iii^	0.93	2.45	3.361 (6)	168
C22—H22···O3^iv^	0.93	2.51	3.364 (4)	153

Symmetry codes: (i) *x*+1/2, −*y*+3/2, *z*+1/2; (ii) *x*, −*y*+1, *z*−1/2; (iii) *x*, *y*+1, *z*; (iv) −*x*+3/2, *y*−1/2, −*z*+1/2.
